# Structured Program for Developing the Psychomotor Skills of Institutionalized Children with Special Educational Needs

**DOI:** 10.3390/children11010102

**Published:** 2024-01-15

**Authors:** Daniel Roșu, Florin Cojanu, Paul-Florinel Vișan, Nicoleta Samarescu, Mariana Augustina Ene, Raul-Ioan Muntean, Vasile Emil Ursu

**Affiliations:** 1 Department of Physical Education and Sport, Faculty of Sciences, Physical Education and Informatics, National University of Science and Technology Politehnica Bucharest, Pitesti University Center, 110040 Pitesti, Romania; 2 Department of Educational Science, Faculty of Education, Social Sciences and Psychology, National University of Science and Technology Politehnica Bucharest, Pitesti University Center, 110040 Pitesti, Romania; 3 Doctoral School Sports Science and Physical Education, National University of Science and Technology Politehnica Bucharest, Pitesti University Center, 110040 Pitesti, Romania; 4 Department of Physical Education and Sport, Faculty of Law and Social Sciences, University “1 Decembrie 1918” of Alba Iulia, 510009 Alba Iulia, Romania

**Keywords:** motor skills assessment, institutionalized education, balance, movement speed

## Abstract

Tailoring motor activities to the unique needs of children with special educational requirements has shown considerable efficacy. Our study aimed to develop a structured program specifically designed to enhance psychomotor abilities, focusing on balance and motor–cognitive skills among 28 students (aged 12–14) from two institutional centers in Romania. The program spanned 36 weeks, with biweekly 30 min sessions. Psychomotor skills were assessed through tests measuring balance, speed of movements, and upper limb motor laterality. Initial and final data were collected for evaluation. A statistical analysis, employing the Kolmogorov–Smirnov and Wilcoxon Z tests, compared the assessments. The results indicated significant improvements in movement speed, with a notable increase in stimulus identification (averaging from approximately 13 to 14) (*p* < 0.05) and reduced processing time (decreasing from about 28.7 to 28 s) (*p* < 0.05). However, while the structured program demonstrated substantial enhancements in specific motor and cognitive–motor skills, it did not yield significant changes in dynamic balance, maintaining values close to 0.9 (*p* > 0.05) for open-eyed balance and 0.88 (*p* > 0.05) for closed-eyed balance. Additionally, an analysis of the processing speed in pulses per second showcased a marginal decline, from approximately 0.46 to 0.45, revealing notable disparities between the initial and final measurements (*p* < 0.05).

## 1. Introduction

Children with special educational needs (SEN) encompass a diverse spectrum of learning difficulties that can range from mild to severe. These difficulties often manifest as challenges in acquiring academic skills, such as reading, writing, comprehension, and mathematical abilities [1,2,3] (Jylänki et al., 2022; Coates, 2011; Yılmaz & Soyer, 2018). Additionally, they might encounter obstacles in processing and retaining information, which can affect their learning pace and their depth of understanding across various subjects [4]. The sources of these challenges can be multifaceted [5]. According to Hsue et al. (2009) [6], some children with SEN might have specific learning disabilities, such as dyslexia (impacting reading), dysgraphia (affecting writing), or dyscalculia (influencing mathematical abilities). Others might face attention-related difficulties, like the attention deficit hyperactivity disorder (ADHD), impacting focus and concentration [7].

Furthermore, sensory processing disorders might contribute to learning difficulties among children with SEN [8,9]. Challenges in processing sensory information—such as difficulties with auditory, visual, tactile, or proprioceptive stimuli—can hinder their ability to effectively interact with their environment and comprehend information presented to them [10,11].

Social and emotional factors can also play a significant role. Children with SEN might encounter challenges in social interactions, communication, and emotional regulation, impacting their overall wellbeing and academic performance. These challenges might result from difficulties in understanding social cues, forming friendships, or managing emotions effectively [12,13].

The combination of these factors often leads to diverse learning profiles among children with SEN, making it essential to tailor interventions and educational strategies to meet their specific needs. These difficulties significantly impact their physical and social development, warranting interventions designed to enhance motor–cognitive abilities [14,15,16,17].

Understanding the challenges faced by children with SEN is fundamental to addressing their complex developmental needs [18,19]. Within institutionalized settings, their involvement in sports activities and team games is pivotal not only for motor skill development but also for socialization, fostering inclusivity and bridging the gap between academic learning and real-life experiences [20,21,22].

Educational programs focusing on psychomotor skill development have emerged as crucial interventions aimed at improving motor skills through structured training involving movement-based games targeting various aspects such as balance, coordination, and overall motor abilities [23,24,25]. Evidence suggests that programs integrating stimuli from multiple sensory systems, such as vestibular, auditory, tactile, and visual systems, play a vital role in enhancing motor–cognitive skills among individuals with SEN [26,27].

Moreover, interventions like adapted sports circuits and specialized exercises have shown effectiveness in improving specific motor skills like balance, coordination, and laterality among individuals with SEN [28,29,30]. Playful activities integrated into psychomotor education programs have been demonstrated to enhance participant motivation, aiding concentration and the successful completion of motor–cognitive tasks [31,32].

A holistic approach to psychomotor education, considering movement as a comprehensive perspective involving perceptual, motor, and sensory activities, holds promise in promoting overall adolescent development, addressing not only motor skills but also social, emotional, and psychological aspects [33]. Complementary interventions like occupational therapy, physiotherapy, and psychological interventions aim to address the broader impact of SEN on adolescent development, encompassing various facets beyond motor skills [34,35].

The intervention implemented in this study aims to address these diverse challenges by focusing on enhancing psychomotor skills—encompassing motor coordination, balance, sensory integration, and overall physical and cognitive development—to empower adolescents with SEN and improve their overall wellbeing and academic performance.

The primary objective of special education programs is to cater to the needs of children requiring specialized education, whose demands cannot be adequately met in traditional programs [36,37,38].

The outlined problem highlights a critical need for targeted interventions that go beyond the conventional approaches, aiming to bridge this developmental gap and enhance the overall psychomotor abilities of adolescents with SEN. This study’s objective is to address this specific problem by implementing and evaluating a structured psychomotor program designed to meet the unique needs of adolescents aged 12 to 14 with SEN, ultimately aiming to mitigate the challenges they face in their motor–cognitive skills and overall development.

To meet this aim, the objectives for our research were as follows:

1. Conducting a comprehensive initial assessment to comprehend the level of motor development and physical condition of the participants.
2. Developing a psychomotor program based on the primary data obtained from the initial assessment.
3. Performing a final evaluation to assess the progress and changes in the psychomotor skills of adolescents with SEN.
4. Comparing the initial and final data through a statistical and mathematical analysis to determine the effectiveness of the program in enhancing psychomotor skills.

## 2. Materials and Methods

### 2.1. Participants

The investigation was carried out at two inclusive education school centers. The participants comprised 28 children categorized with SEN (mean age 13.69 ± 0.8 years, height 161.7 ± 6.7 cm, weight 54.9 ± 7.3 kg, and BMI 20.5 kg/m^2^). Out of these, 12 subjects attended the first institutional center, while 16 were enrolled at the second one. We employed a conventional sampling approach, taking into account the age and diversity of individual needs and abilities among children with SEN, to ensure the representativeness and relevance of the obtained results within this study. Parental consent, as per the principles outlined in the Declaration of Helsinki, was a prerequisite and was obtained before the commencement of this study. The Ethics Committee of the Doctoral School of Physical Education and Sport Science (ID: 04/21.07.2023), University of Pitesti, Romania, granted approval for this research.

### 2.2. Research Tools

The examinations were conducted employing specialized equipment, devices, and their corresponding software modules. These facilitated the recording, processing, and consolidation of individualized data for each subject.

The Sensamove balance miniboard was employed to assess static coordination in this study. This specialized tool allowed for the precise evaluation of an individual’s ability to maintain stability and control in static positions. Through its sensitive sensors and calibrated surface, the miniboard facilitated the measurement and analysis of the balance and coordination performance during static tasks. Its utilization enabled the collection of detailed data concerning the participants’ static coordination capabilities, contributing to a comprehensive assessment within the research [39].

This study utilized the Optojump Next optical system, a high-tech tool for analyzing perceptual–motor coordination. This system, equipped with advanced sensors and software, precisely captured and assessed various aspects of human movement. Specifically, it evaluated the synchronization between sensory perception and motor responses, providing crucial insights. It was instrumental in measuring dynamic balance under different conditions in our research, enhancing our understanding of the participants’ perceptual–motor coordination [40].

The Witty SEM Intelligent semaphores were employed to quantify movement speed in this study. This research tool consists of specialized equipment designed to measure and assess the velocity and agility of various physical movements. It operates by capturing and analyzing the time required for specific movements or series of actions, providing precise measurements of movement speed. Through its utilization, this tool contributed valuable insights to the assessment of movement speed, enhancing this study’s understanding of the participants’ kinetic capabilities [41,42].

### 2.3. Procedure of the Intervention

In the first stage (September 2022), the psychological records of the subjects were analyzed. The records were filled in by the psychologists of the institutional centers for each individual subject, throughout the institutionalization period.

In the second stage (October 2022), the subjects went through a series of tests meant to provide specific information. The tests focused on the manifestation of some components in the sphere of psychomotricity and motor skills.

Following the preliminary research phase, we processed and analyzed the gathered data, and the resulting conclusions guided the selection process of psychomotor tools and the establishment of action systems within the segments of the psychomotor program.

In the third stage, the participants were integrated into an experimental group, benefiting from an adaptive psychomotor education program. The program comprised six action systems, each sustained for two weeks and repeated three times upon the completion of a cycle (November 2022 to July 2023). Table 1 illustrates the dynamics and synthesized content of these action systems.

In outlining strategies, the focus was on enhancing balance, coordination, laterality, and movement speed. Considering the corresponding age peculiarities (12–14 years old) as outlined in Table 1, objectives correlated with specific content were established. According to Patrascan and Stefanica (2019) [43], there exists a close correlation and interdependence between the physical development of students with SEN and their mental capacity, assimilation, and utilization of knowledge. Huotari et al. (2011) [44] delved into how tailored exercise routines impacted the physical wellbeing of children facing intellectual disabilities (ID). Additional research emphasized the advantages of engaging in exercise to enhance skill-related fitness components (SRF) among young individuals with intellectual disabilities (ID) [45].

Table 1 presents the temporal dynamics of systems of actions through psychomotor means and their corresponding objectives. These actions were grounded in engaging activities designed to enhance the participants’ balance and motor–cognitive skills.

As a result, in Table 1, our aim was to enhance, through elaborated action systems, the following psychomotor abilities: dynamic balance, static and dynamic coordination, spatial orientation, perceptual–motor coordination, laterality, movement speed, and rhythm.

The first step was identifying the components of the educational strategy (objectives, content, methods, resources, material base, forms of activity organization, and evaluation) for its optimal development and implementation in practice. An essential role in this process was played by the feedback obtained throughout the preparation step, contributing to adjusting the educational strategy to emerging conditions and situations. There were three cycles, each comprising six action systems. At the end of each cycle, an evaluation of the results obtained and the problematic aspects arising in practice was conducted. The action systems are not fixed tools but rather adaptable and modifiable tools that evolved over the period of working with children with SEN.

The second step involved the development and application of the psychomotor action systems presented in Table 1. The action systems were implemented based on the following practical applications:

1. Organizing them according to the components of educational activities, in various forms, such as practical courses, dynamic games, relays, and contests, designed through psychomotor means, through tasks and psychomotor actions adapted to children with SEN.
2. Methods used—exercise and collective game method, competitive methods, explanations, presentations, conversations, and demonstrations.
3. Specific means and materials—gymnastic benches, horse apparatus, rings, gymnastic vaults, trampoline, parallel bars, hoops, cones, small, medium, and large mats, cubes, rods, medicine balls, coordination balls, Bossu balls, small gates of 1 m, etc.

### 2.4. Statistical Analysis

In the fourth stage (August 2023 to September 2023), the statistical–mathematical analysis aimed to compare the results of the initial and final assessments was conducted. In this analysis, several statistical tests were employed:

1. Investigation of the normality of distributions—this examines whether the data follow a normal distribution or not. It was evaluated using the Kolmogorov–Smirnov test.
2. Significance of differences between distributions—measured through a nonparametric test, the Wilcoxon Z test, utilized to ascertain significant differences between two data sets or two sets of measurements, particularly when the data are not normally distributed.

For each specific measurement (such as static balance, movement speed, dynamic balance, etc.), statistical tests were conducted to compare the initial (I) and final (F) test outcomes. The t-tests employed in these comparisons include independent t-tests as well as nonparametric tests like the Wilcoxon Z test. The test results are presented with mean values, standard deviation, the number of subjects, and Z-values, with significance expressed through *p*-values (probability).

## 3. Results

Table 2 presents the initial and final results obtained from tests targeting various components of psychomotor skills, encompassing static coordination, perceptual–motor coordination (involving spatial perception, rhythm, and self-movement perception).

In Table 3, the normality of distributions is examined using the Kolmogorov–Smirnov test. The Kolmogorov–Smirnov statistic values are presented for different measurements, along with their associated *p*-values (static balance %, movement speed, and dynamic balance). The *p*-values obtained from the Kolmogorov–Smirnov tests suggest that the distributions differ from normal distributions in most cases, (*p* < 0.05), except for the static balance %, movement speed reaction time, and dynamic balance (eyes open–left leg) measurements, where the *p*-values are greater than 0.05 (*p* > 0.05), indicating that the distributions of these measurements are not significantly different from a normal distribution.

Table 4, outlining the significance of the differences between distributions using the Wilcoxon Z test, examines significant differences between distributions using the Wilcoxon Z test. The comparison between the initial (I) and final (F) distributions are as follows:

1. For most measurements, there are significant differences between the initial and final distributions (*p* < 0.05), such as for the movement speed number of stimuli, movement speed reaction time, processing speed pulses/sec, and dynamic balance measurements. This suggests that these measurements underwent significant changes between the two testing moments.
2. In the case of static balance and certain dynamic balance measurements, there are no significant differences between the initial and final distributions (*p* > 0.05), indicating stability in these measurements during the testing period.

**Table 4:** Significance of differences between distributions using a nonparametric test, the Wilcoxon Z test.

Measurements	Test ^1^	Mean	Std. Deviation	N	Z	Sig. (2 Tailed)	*p*
Static Balance	I	60.67	23.347	28	0.894	0.371	*p* > 0.05
	F	61.29	22.346	28			
Movement speed—number of stimuli	I	13.46	3.636	28	2.504	0.012	*p* < 0.05
	F	13.92	3.420	28			
Movement speed—reaction time	I	28.78	0.976	28	3.286	0.001	*p* < 0.05
	F	27.97	1.305	28			
Processing speed pulses/sec.	I	0.46	0.123	28	2.423	0.015	*p* < 0.05
	F	0.45	0.144	28			
Dynamic balance—eyes open, left leg	I	0.94	0.449	28	1.480	0.139	*p* > 0.05
	F	0.91	0.421	28			
Dynamic balance—eyes open, straight leg	I	0.92	0.282	28	1.598	0.110	*p* > 0.05
	F	0.90	0.290	28			
Dynamic balance—eyes closed, left leg	I	0.91	0.330	28	1.936	0.53	*p* > 0.05
	F	0.88	0.370	28			
Dynamic balance—eyes closed, straight leg	I	0.92	0.348	28	1.754	0.80	*p* > 0.05
	F	0.89	0.326	28			

^1^ I = Initial testing; F = Final testing.

## 4. Discussion

The essential contribution within our research lies in the development and implementation of an optimal psychomotor program tailored to children with SEN, adapted to each individual’s complex characteristics. By practicing physical education and sports, thus strengthening their physical vigor and health, students create the essential conditions required for intense inclusive activities and beneficial social engagement [46].

The findings of our research demonstrated improvements in strength, balance, and fat-free mass, accompanied by reductions in fat mass and waist circumference. Consequently, the overall quality of life for the studied adolescents with special educational needs and intellectual disabilities experienced notable enhancements [47]. Within the specialized literature, attention has been directed towards literacy instruction methods tailored to young individuals with cognitive disabilities. Researchers are exploring new approaches aimed at enhancing this domain. Moreover, specialists have scrutinized the consequences of an unhealthy diet among children and adolescents. Such dietary patterns might contribute to overweight conditions and cardio-metabolic risks and could potentially impair cognition and academic performance [48]. Studies have explored high school students’ perceptions of adolescents with special educational needs with a focus on the imperative need to improve both students’ and teachers’ attitudes toward these individuals. Special educational needs encompass conditions like Tourette syndrome. Experts clarify that the Tourette syndrome is a neuro-behavioral and psychiatric disorder, constituting a disability. Consequently, adolescents with Tourette syndrome ought to be understood rather than penalized. It is crucial for students to cultivate acceptance toward adolescents with special educational needs [49].

The verification process aimed to evaluate the intervention’s impact on diverse aspects of the subjects’ psychomotor abilities. The outcomes reveal the following observations, as depicted in Table 3 and Table 4:

1. Regarding static balance, there were no substantial differences observed between the initial and final measurements, indicating consistent levels maintained at an average of approximately 60–61%.
2. In terms of movement speed, notable variances emerged between the initial and final measurements, denoting a distinct augmentation. The identified stimuli increased significantly from an average of about 13 to 14, while Movement speed reaction time decreased from approximately 28.7 to 28 s.
3. The analysis of the processing speed showed a slight decrease, from around 0.46 to 0.45 pulses per second, indicating substantial differences between the original and final observations.
4. The dynamic balance assessments conducted with eyes open and closed revealed no significant disparities between the initial and final measurements. The values remained relatively stable, averaging at 0.9 for open-eyed balance and 0.88 for closed-eyed balance.

Interestingly, the assessments revealed that static balance (60–61%) remained consistent throughout the intervention, suggesting stability in this aspect among the participants. However, the slight decrease in the processing speed in pulses per second (0.46 to 0.45) indicates some alterations in this parameter despite not being as pronounced as the improvements seen in movement speed number of stimuli (from about 13 to 14).

The tests movement speed number of stimuli, movement speed reaction time, processing speed pulses/sec., dynamic balance (eyes open, left leg), dynamic balance (eyes open, straight leg), dynamic balance (eyes closed, left leg), and dynamic balance (eyes closed, straight leg) showed significant changes (*p* < 0.05) between the initial and final tests.

Other tests such as static balance, dynamic balance (eyes open, left leg), dynamic balance (eyes open, straight leg), dynamic balance (eyes closed, left leg), and dynamic balance (eyes closed, straight leg) did not show significant changes (*p* > 0.05) between the initial and final tests.

Therefore, overall, psychomotor skills, measured in certain aspects through specific tests, significantly improved following the psychomotor skills development program. This is supported by the *p* < 0.05 values in the respective tests.

These data suggest that the intervention had a significant impact on movement speed reaction time and certain aspects thereof, while no notable changes were observed in the subjects’ static or dynamic balance. It is important to note that these results provide insight into the subjects’ progression following our intervention, offering crucial information regarding how it might influence their psychomotor profiles. Ultimately, while the intervention showcased notable enhancements in movement speed, the findings underscore the program’s limitations in influencing certain elements of psychomotor skills, particularly static and dynamic balance.

The specialized literature has focused too heavily on literacy instruction for young people with cognitive disabilities. In this regard, Farrokhian et al. (2021) [50] studied functional training’s impact on balance and flexibility in female students with an intellectual disability. Thirty participants were divided into experimental and control groups. After 15 sessions, those in the experimental group showed significant improvements in their static and dynamic balance and flexibility. The study recommended integrating functional training into programs for students with intellectual disabilities. Hsu (2016) [51] selected 24 students with disabilities, splitting them into Wii Fit and physical education groups. After eight weeks, the Wii Fit group displayed notable improvements in various parameters, while the physical education group mainly improved in their speed movement index. Van Biesen et al. (2017) [52] explored the link between intelligence and reaction time in athletes with mild to moderate intellectual impairment. The study involved 103 individuals with intellectual impairment, testing whether intelligence and reaction time were correlated. A comparison group of 103 tertiary education students was also recruited. The study showed that reaction time increased with task complexity, more so in athletes with intellectual impairment than in the comparison group. This research sets a precedent, highlighting that impaired intelligence impacts reaction time, especially in tasks with high cognitive loads.

## 5. Limitations and Strengths

Regarding limitations, this study focused on specific aspects of psychomotor abilities, overlooking other relevant elements which could have contributed to a more comprehensive understanding. The intervention duration of 36 weeks, while significant, might have posed a constraint given its relatively short timeframe for developing psychomotor abilities, particularly among children with SEN, who often exhibit a slower pace of development. In terms of strengths, the personalized approach to the psychomotor program for children with special educational needs stands as a significant aspect, addressing the complex and individualized needs of the participants. Moreover, this study demonstrates notable improvements in specific aspects of psychomotor abilities, particularly in movement speed and reaction to stimuli. This study’s rationale is supported by a diverse range of credible references from the specialized literature, thereby consolidating its relevance and argumentation within the academic domain.

## 6. Conclusions

This study revealed significant insights into our intervention’s effects on diverse psychomotor abilities among the participants. Notable enhancements were observed in movement speed, evidenced by heightened stimulus identification and decreased processing time. Conversely, static balance remained stable, and there were no substantial alterations in dynamic balance between the initial and final assessments. These findings underscore the intricate nature of addressing psychomotor abilities in children with SEN, emphasizing the need for more customized interventions to comprehensively tackle these varied skill sets.

## Figures and Tables

**Table 1:** The dynamics and synthesized content of action systems.

No.	Description of Psychomotor Program ^1^	Objectives of Psychomotor Actions Reflecting the Program
1	Systems of action through psychomotor means for dynamic balance and static and dynamic coordination Implementation Period: 2 weeks × 3 (Weeks 1–2, 13–14, 25–26) Practice Duration: 30 min	- Execute a sequence of six steps marked at first glance in three attempts; - Perform motor actions with progressively increased complexity and effort; - Maintain a tempo in executing an exercise from the running school by means of a ladder/circle systematically applied on the ground; - Enhance the capacity to perform motor acts with increased speed indices.
2	Systems of action through psychomotor means for improving dynamic balance, spatial orientation, and perceptual–motor coordination Implementation Period: 2 weeks × 3 (Weeks 3–4, 15–16, 27–28) Practice Duration: 30 min	- Execute the correct sequence of positioning and start from the bottom when speed-running; - Perform motor actions with progressively increased complexity and effort specific to speed-running; - Maintain a tempo in executing an exercise from the running school by means of a ladder/circle systematically applied on the ground, synchronizing running steps through marked zones on the ground; - Enhance the capacity to perform motor acts with increased speed indices.
3	Systems of action through psychomotor means for spatial orientation education, dynamic balance, and laterality Implementation Period: 2 weeks × 3 (Weeks 5–6, 17–18, 29–30) Practice Duration: 30 min	- Master the basic mechanism of endurance running steps as well as the capacity for adaptation to effort; - Perform motor actions with progressively increased complexity and effort; - Enhance the capacity to perform motor acts amidst the effort of age-specific endurance and individual motor abilities.
4	Systems of action through psychomotor means for educating dynamic balance and laterality Implementation Period: 2 weeks × 3 (Weeks 7–8, 19–20, 31–32) Practice Duration: 30 min	- Maintain balance in a vertical position with a 1.5 m stick while moving on various fixed/mobile/balance surfaces; - Execute a sequence of three turns of 90°, 180°, and 360° on a suspended balance surface at 1.6 m (protected surface provided using mattresses); - Demonstrate the ability to grasp and throw a basketball/handball/volleyball/sponge ball while maintaining balance while moving on an inverted bench positioned on sticks, with freedom of movement between two box lids; - Consistently pass the mini-basketball sideways with a partner while moving on a balance surface at a height of 1.5 m.
5	Systems of action through psychomotor means for educating coordination, movement speed, rhythm, and laterality Implementation Period: 2 weeks × 3 (Weeks 9–10, 21–22, 33–34) Practice Duration: 30 min	- Execute a sequence of six steps marked with circles on the ground, simultaneously catching the ball passed from the side and pushing it from the chest to a marked area at 10 m in a “dart” system; - Perform motor actions with progressively increased complexity by catching and throwing balls while moving; - Execute eight passes while catching the mini-basketball/soccer/volleyball/handball/sponge ball, while running through systematically arranged circles in a zigzag pattern interspersed with “skipping” exercises.
6	Systems of action through psychomotor means for educating coordination, movement speed, and laterality Implementation Period: 2 weeks × 3 (Weeks 11–12, 23–24, 35–36) Practice Duration: 30 min	- Execute a sequence of three turns of 90°, 180°, and 360° on a suspended balance surface at 1.6 m (protected surface provided by mattresses); - Demonstrate the ability to grasp and throw a basketball/handball/volleyball/sponge ball while maintaining balance while moving on an inverted bench positioned on sticks, with freedom of movement between two box lids.

^1^ At the conclusion of each motor activity undertaken by individual students, upon completion of routes, relays, and psychomotor courses, the students will have a 10–15 s period facilitated by the specialized teaching staff to complete worksheets. During this time, they will be required to provide rapid non-verbal responses solely through written gestures or indicated reactions on the work board positioned at the finish line.

**Table 2:** Psychomotor profile of the subjects before and after intervention.

No	Static Balance (%)		Movement Speed						Dynamic Balance with Open-Eyes				Dynamic Balance with Closed-Eyes			
			Identified Stimuli (no)		Reaction Time (s)		Processing Speed (Pulses/sec.)		Contact Time (ms)				Contact Time (ms)			
									Left Foot		Right Foot		Left Foot		Right Foot	
I/F ^1^	I	F	I	F	I	F	I	F	I	F	I	F	I	F	I	F
1	71	75	10	10	28.74	28.12	0.35	0.31	0.83	0.84	0.81	0.79	0.80	0.76	0.79	0.77
2	60	60	9	11	27.32	27.63	0.33	0.28	0.72	0.70	0.73	0.70	0.69	0.54	0.68	0.56
3	42	50	9	10	27.51	25.56	0.33	0.27	0.69	0.66	0.70	0.62	0.71	0.70	0.72	0.68
4	68	72	9	9	28.24	27.32	0.32	0.28	0.75	0.70	0.58	0.56	0.83	0.81	0.70	0.69
5	35	34	9	9	28.69	28.12	0.31	0.30	0.94	0.88	0.90	0.92	0.96	0.98	0.92	0.88
6	32	39	9	11	28.34	26.63	0.32	0.28	0.85	0.82	0.74	0.74	0.72	0.70	0.73	0.67
7	58	58	9	9	29.65	25.56	0.30	0.30	2.83	2.69	1.64	1.58	1.13	1.20	1.20	1.24
8	18	19	9	10	29.84	29.56	0.30	0.28	0.84	0.87	0.87	0.79	0.91	0.88	0.86	0.75
9	11	12	9	9	29.33	28.13	0.31	0.27	1.82	1.79	1.55	1.54	2.34	2.44	2.45	2.21
10	79	79	8	9	29.42	27.13	0.27	0.27	0.88	0.78	0.83	0.77	0.69	0.75	0.69	0.73
11	83	83	15	14	28.41	29.41	0.53	0.50	0.98	0.92	1.04	0.98	0.94	0.85	0.85	0.78
12	35	36	15	15	29.34	30.78	0.51	0.47	0.71	0.73	0.69	0.59	0.69	0.68	0.67	0.75
13	17	17	14	15	29.37	29.19	0.48	0.45	0.83	0.80	0.91	0.95	0.92	0.88	0.92	0.86
14	69	68	13	14	25.19	25.15	0.52	0.47	0.89	0.82	0.92	0.92	0.83	0.75	0.89	0.85
15	74	74	15	15	29.08	29.20	0.52	0.52	1.42	1.44	1.25	1.23	1.36	1.48	1.36	1.33
16	79	79	18	18	28.64	27.32	0.63	0.60	0.60	0.74	1.42	1.40	0.83	0.78	0.83	0.77
17	86	85	14	16	28.92	28.12	0.48	0.49	0.86	0.66	1.30	1.38	0.81	0.83	0.86	0.94
18	73	73	18	18	28.46	26.63	0.63	0.62	0.69	0.82	0.70	0.72	0.70	0.66	0.70	0.62
19	44	46	17	18	29.78	27.56	0.57	0.58	0.78	0.85	0.74	0.65	0.74	0.66	0.74	0.63
20	79	77	18	18	29.72	29.72	0.61	0.61	0.80	0.71	0.84	0.79	0.85	0.75	0.87	0.99
21	90	88	18	17	29.90	28.13	0.60	0.53	0.66	0.65	0.64	0.64	1.00	1.03	1.02	1.04
22	55	54	15	16	29.21	28.13	0.51	0.66	0.73	0.70	0.76	0.77	0.80	0.73	0.96	0.84
23	81	78	17	17	29.13	29.13	0.58	0.58	0.65	0.66	0.73	0.71	0.68	0.56	0.77	0.65
24	77	76	18	17	29.62	28.12	0.61	0.75	0.80	0.88	0.89	0.90	0.72	0.63	0.80	0.75
25	76	79	15	17	29.18	28.63	0.51	0.48	0.80	0.78	0.72	0.71	0.82	0.79	1.03	1.09
26	48	50	15	15	28.45	28.45	0.53	0.53	0.94	0.84	0.75	0.90	0.97	0.88	0.79	0.90
27	93	89	16	17	28.19	27.56	0.57	0.55	1.30	0.99	1.27	1.29	1.33	1.30	1.31	1.30
28	66	66	16	16	28.33	28.33	0.56	0.56	0.81	0.93	0.85	0.88	0.80	0.86	0.85	0.87

^1^ I = Initial testing; F = Final testing.

**Table 3:** Investigation of normality of distributions.

Measurements	I/F ^1^	df	Statistic Kolmogorov–Smirnov	Sig. KolgSmirn	Interpretation *p*	Finding/ Status of Distributions	Statistic Test
Static Balance %	I	28	0.162	0.059	*p* > 0.05	A normal and a nonnormal distribution	Wilcoxon Z
	F	28	0.184	0.016	*p* < 0.05		
Movement speed—number of stimuli	I	28	0.212	0.002	*p* < 0.05	Two abnormal distributions	Wilcoxon Z
	F	28	0.194	0.008	*p* < 0.05		
Movement speed—reaction time	I	28	0.164	0.053	*p* > 0.05	A normal and a nonnormal distribution	Wilcoxon Z
	F	28	0.151	0.101	*p* < 0.05		
Processing speed pulses/sec.	I	28	0.206	0.004	*p* < 0.05	Two abnormal distributions	Wilcoxon Z
	F	28	0.202	0.005	*p* < 0.05		
Dynamic balance—eyes open, left leg	I	28	0.325	0.001	*p* < 0.05	Two abnormal distributions	Wilcoxon Z
	F	28	0.344	0.001	*p* < 0.05		
Dynamic balance—eyes open, straight leg	I	28	0.248	0.001	*p* < 0.05	Two abnormal distributions	Wilcoxon Z
	F	28	0.198	0.006	*p* < 0.05		
Dynamic balance—eyes closed, left leg	I	28	0.257	0.001	*p* < 0.05	Two abnormal distributions	Wilcoxon Z
	F	28	0.294	0.001	*p* < 0.05		
Dynamic balance—eyes closed, straight leg	I	28	0.262	0.001	*p* < 0.05	Two abnormal distributions	Wilcoxon Z
	F	28	0.216	0.002	*p* < 0.05		

^1^ I = Initial testing; F = Final testing.

## Data Availability

Data available on request due to restrictions e.g., privacy or ethical. The data presented in this study are available on request from the corresponding author. The data are not publicly available due to confidentiality.
